# RNA-seq transcriptomic analysis of adult zebrafish inner ear hair cells

**DOI:** 10.1038/sdata.2018.5

**Published:** 2018-02-06

**Authors:** Cody L. Barta, Huizhan Liu, Lei Chen, Kimberlee P. Giffen, Yi Li, Kenneth L. Kramer, Kirk W. Beisel, David Z. He

**Affiliations:** 1 Department of Biomedical Sciences, Creighton University School of Medicine, Omaha, Nebraska 68178, USA; 2 Chongqing Academy of Animal Science, Chongqing 402460, China; 3 Department of Otorhinolaryngology-Head and Neck Surgery, Beijing Tonren Hospital, Capital Medical University, Beijing 100730, China

**Keywords:** RNA sequencing, Zebrafish, Hair cell, Transcriptomics

## Abstract

Although hair cells are the sensory receptors of the auditory and vestibular systems in the ears of all vertebrates, hair cell properties are different between non-mammalian vertebrates and mammals. To understand the basic biological properties of hair cells from non-mammalian vertebrates, we examined the transcriptome of adult zebrafish auditory and vestibular hair cells. GFP-labeled hair cells were isolated from inner-ear sensory epithelia of a *pou4f3* promoter-driven GAP-GFP line of transgenic zebrafish. One thousand hair cells and 1,000 non-sensory surrounding cells (nsSCs) were separately collected for each biological replicate, using the suction pipette technique. RNA sequencing of three biological replicates for the two cell types was performed and analyzed. Comparisons between hair cells and nsSCs allow identification of enriched genes in hair cells, which may underlie hair cell specialization. Our dataset provides an extensive resource for understanding the molecular mechanisms underlying morphology, function, and pathology of adult zebrafish hair cells. It also establishes a framework for future characterization of genes expressed in hair cells and the study of hair cell evolution.

## Background & Summary

Hair cells are the sensory receptors of the auditory and vestibular systems in the ears of all vertebrates. Hair cells transduce mechanical stimuli, such as movement in their environment, into electrical activity^1^. The site of such mechanoelectrical transduction is the hair bundle, the hallmark of all hair cells. In addition to the apical stereocilia bundle, hair cells also contain structural and functional specializations in the basolateral and synaptic membranes, which are responsible for electrical activities and synaptic transmission^2–4^.

Although hair cells have some common properties, hair cells in non-mammalian vertebrates are substantially different from their mammalian counterpart. For example, hair cells of non-mammals are electrically tuned to specific frequencies and possess an active process in the stereocilia bundle to amplify sound signals^5,6^. Mammalian hair cells, in contrast, are not electrically tuned^7^. Mammals enhance their auditory sensitivity and frequency selectivity by outer hair cell electromotility^8–10^, a mammalian innovation. Hair cells from non-mammalian vertebrates such as bullfrog and chicken, are able to spontaneously repair and regenerate their damaged or lost stereocilia bundles^11–13^. In contrast, mammalian cochlear hair cells no longer retain that capability^14^. To understand the molecular mechanism underlying their differences, analysing transcriptomes of hair cells is an important first step. The transcriptome represents all the RNA transcripts in a cell and can vary depending on cell type, physiological condition, and development stage^15^. The adult zebrafish hair cell transcriptome was examined by the first generation of DNA microarray technique consisting of 15,000 transcript probes^16^. That study was completed before a complete zebrafish reference genome became available^17^. Transcriptomic analysis using RNA-seq allows assessment of the quantitative expression over 31,000 genes of the GRCz10 zebrafish genome, as well as possible identification of new splice variants. Several recent studies examined hair cell transcriptomes from zebrafish larval with RNA-seq^18–21^. Those studies have led to the identification of some hair cell-specific genes. However, since hair cells in those studies were obtained from zebrafish larvae, whose gene expression profile is known to be different from that of adult hair cells, we examined the inner ear hair cell-specific transcriptome of adult zebrafish using RNA-seq.

Our goal of sequencing the transcriptome of pristine populations of adult zebrafish hair cells was accomplished by using GFP-labeled hair cells isolated from the utricle, saccule, and lagena, the three inner-ear sensory epithelia of a *pou4f3* promoter-driven GAP-GFP line of transgenic zebrafish^22^. In addition to GFP-labeling, hair cell identification was further aided by the visible morphological feature of all hair cells, the stereocilia bundle, to exclude any ambiguous cells from collection. A suction pipette was used to individually collect hair cells^23,24^. This technique has some advantages over the fluorescence-activated cell sorting (FACS) technique^25^. In our study, cells were individually collected based on the presence of both GFP expression and stereocilia bundles. Thus, cell identity was unambiguous and potential contamination by other cell types was mitigated. Another advantage is that the average time for collection of 300 to 350 hair cells from each zebrafish after hair cells were isolated was less than 50 min. Because cells were maintained in cold solution during collection, and individually collected cells were immediately fixed in RNA*Later* solution, this shorter time of cell sorting allows isolation of high quality RNA and minimizes transcriptomic changes that may occur during FACS, which may take up to a few hours^25^.

**Figure 1:**
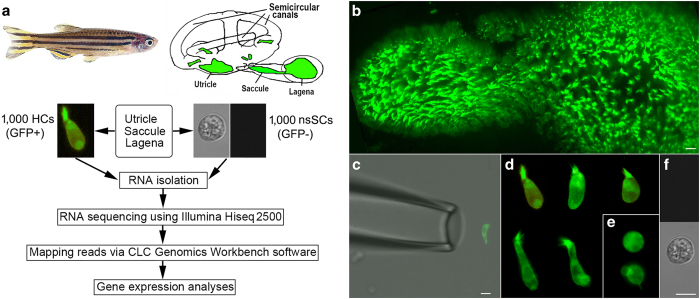
Study design workflow for cell isolation and collection for transcriptome analysis of GFP-positive hair cells (we used HCs in all figures) and GFP-negative nsSCs isolated from adult zebrafish inner ear. Schematic drawing of zebrafish is modified from Fig. 1 of a previous publication^37^ (with permission from Frontier in Cellular Neuroscience). (**a**) Workflow of experimental design for RNA-seq and transcriptome analysis for 1,000 individually collected hair cells and nsSCs. (**b**) GFP-expressing hair cells in saccule and lagena of zebrafish inner ear. (**c**) Suction pipette technique used to manually collect individual hair cells and nsSCs. (**d**) Examples of GFP-expressing hair cells. Only those cells that had both GFP expression and stereocilia bundles were selected. (**e**) Example of GFP-expressing cells without visible stereocilia bundles. The identify of these cells was unknown, so they were not collected. (**f**) An example of a nsSC with no GFP expression. An equal number of nsSCs was individually collected for comparison with hair cells. Bars: 20 μm (**b**), 10 μm (**c**), and 10 μm (**d**–**f**).

Here, we describe transcriptome-wide profiling of hair cells and non-sensory surrounding cells (nsSCs) from the adult zebrafish inner ear. Three biological replicates, each containing 1,000 individually collected hair cells, were prepared. Each of our three control samples consisted of 1,000 isolated nsSCs from the inner ear that did not exhibit fluorescence and stereocilia bundles. An overview of the study design is depicted in Fig. 1a. Careful and stringent technical design at each experimental stage has allowed generation of a high-quality RNA-seq dataset which has tremendous value for future characterization of all genes expressed in zebrafish hair cells. Our dataset is also expected to provide valuable information for the study of hair cell regeneration and evolution. Hair cell-specific transcriptomes from mouse cochleae and vestibule have been analyzed^20,24,26–28^. Thus, the present dataset is also useful for comparative studies of hair cells between zebrafish and mouse.

## Methods

### Hair cell isolation and collection

Adult female transgenic *Tg(pou4f3:GAP43-GFP)s273t* zebrafish^22^ at 11 to 13 months of age were used for the study. Animals were euthanized by submersion in ice water (0–4 °C) for ten minutes after cessation of opercular movement. The utricle, saccule, and lagena were isolated using a method described by Liang and Burgess^29^. Hair cells in the inner ear structures of this transgenic zebrafish line express GFP and an example of GFP-expressing hair cells in the isolated saccule and lagena is shown in Fig. 1b. The inner ear tissues then underwent an enzymatic digestion at room temperature for 20 min in the medium containing 1 ml of L-15 medium and 1 mg of Collagenase IV (Sigma-Aldrich, St. Louis, MO, USA). The inner ear tissues were transferred to Leibovitz’s L-15 medium at 300 mOsm, 7.35 pH. Hair cells and nsSCs were separated by gentle trituration. The chamber (with inlet and outlet), placed on the stage of an Olympus inverted microscopy with fluorescence capability, was perfused with fresh L-15 medium to wash out debris for 5 min. To collect solitary cells, the suction pipette technique was used^19,20^. Two pickup pipettes with a diameter of ~30 μm were used to separately pick up GFP-positive hair cells and GFP-negative nsSCs. The pipette was fabricated from 1.5 mm thin-wall glass tubing pulled by a two-stage electrode puller. The pickup pipettes were mounted in two separate electrode holders mounted on two Leitz 3-D micromanipulators (Leitz, Germany). By moving the pickup pipette and the stage of the microscope, cells were positioned near the tip of the pipette. The suction port of the pipette holder was connected to a micrometer-driven syringe to provide positive or negative pressure to draw in or expel the cells. An image of a GFP-positive hair cell before being drawn into a pickup pipette is shown in Fig. 1c a video showing a mouse outer hair cell being drawing into a pickup pipette is provided in Supplementary Video 1 (Data Citation 1). After ~10 to 20 cells were drawn into the pipette, the pipette was lifted out from the bath and transferred to a microcentrifuge tube containing 50 μl RNA*later* (Thermo Fisher Scientific, Waltham, MA). Cells were expelled from the pipette by applying positive pressure. We repeated this step until approximately 300 to 350 hair cells and nsSCs were collected from each zebrafish. 1,000 hair cells were collected from approximately four zebrafish for each of the three samples. 15 zebrafish were used for the collection of three biological samples of hair cells and three biological samples of nsSCs. During cell collection, two pickup pipettes were used, each designated for only one cell type, to prevent contamination. To obtain highly specific hair cells, several steps were taken to avoid contamination by other cell types. First, each cell being collected was identified under direct visual observation. Since hair cells used in our study expressed GFP, presence of both GFP and the stereocilia bundle were used to identify hair cells (Fig. 1d). Any ambiguous cells that exhibited only GFP expression without the visible stereocilia bundle (Fig. 1e) were excluded. Second, only solitary cells not attached to any other cell types were collected. Finally, we were careful about the suction pressure applied to the pipette to avoid drawing unwanted cells. The suction pipette (to deposit hair cells) was withdrawn only when the pressure was balanced and no more fluid or cells were being drawn into the pipette. 1,000 nsSCs lacking GFP expression and stereocilia (Fig. 1f) from the inner ear epithelium were also collected for each of the three samples using the same procedures.

### RNA isolation, amplification

Total RNA, including small RNAs (>~18 nucleotides), from approximately 1,000 each cell type separately suspended in RNA*Later* (~100 μl in total volume), were extracted and purified using the Qiagen miRNeasy mini plus Kit (Qiagen Sciences Inc, Germantown, MD). On-column DNase digestion was performed to eliminate DNA contamination in the collected RNA. After purification, the quality and quantity of RNA was determined using an Agilent 2,100 BioAnalyzer (Agilent Technologies, Santa Clara, CA) and compared to examples of pure RNA results found in the Agilent 2,100 Bioanalyzer 2,100 Expert User’s Guide. Total RNA from each sample ranged from 5 to 10 ng/μl. These purified RNA samples were reverse transcribed into cDNA and amplified using the SMART-Seq V4 Ultra Low Input RNA kit (Clontech Laboratories, Inc., Mountain View, CA) to prepare the samples for RNA sequencing.

### RNA-sequencing and bioinformatic analyses

Genome-wide transcriptome libraries were produced from triplicate biological replicates. Libraries were prepared using the SMART-Seq V4 Ultra Low Input RNA kit (Clontech) to generate cDNA in combination with the Nextera Library preparation kit (Illumina, Inc., San Diego, CA). Library size and concentration were assessed using an Agilent 2,100 Bioanalyzer and a Qubit fluorometer (Invitrogen) to ensure the inserts were the appropriate size and determine concentration prior to sequencing. Transcriptome libraries were sequenced using the HiSeq 2,500 Sequencing System (Illumina). Libraries were multiplexed and three samples per 100 bp paired-end lane were sequenced, generating approximately 100 million reads per sample. The cut-off for RNA integrity number was set at the value of 9. The files from the multiplexed RNA-seq samples were demulitplexed and fastq files representing each library and quality control data were generated.

### Code availability and bioinformatics analyses

No custom code was used in these analyses. CLC Genomics Workbench software (CLC bio, Waltham, MA, USA) and UCSC genome were used to map the reads to the GRCz10 zebrafish genome and generate gene expression values in the normalized form of reads per kilobase of transcript per million mapped reads (RPKM) values. Reads were mapped to exonic, intronic, and intergenic sections of the genome. Gene expression estimates were derived from the mapped reads using HTSeq count^30^. Expression data was exported from CLC Genomics Workbench in the form of Microsoft Excel Spreadsheets. Clustering analyses were carried out using one of the following algorithms: CLICK, K-means and SOM, which are implemented in the EXPANDER integrative package for analysis of gene expression. The differentially expressed genes and clusters were examined for enrichment of known biological processes using DAVID^31^ and Ingenuity IPA program (www.ingenuity.com). Entrez Gene, HGNC, OMIM, and Ensembl database were used for verification, reference, and analyses. Additional resources such as the gEAR (http://umgear.org/index.html) and SHIELD (https://shield.hms.harvard.edu/index.html)^32^ websites were also used for transcriptome profile comparisons.

### Quantitative reverse transcription PCR (qPCR)

Quantitative-PCR experiments were run on an Applied Biosystems 7,500 Fast Real-Time PCR system. Ten microliters of Powerup SYBR Green Master Mix (Thermo Fisher Scientific, Waltham, MA, USA) was used in each 20 microliter reaction. Primer concentrations were 450 nM. The original cDNA samples were diluted twenty-fold with two microliters for every reaction. The fast thermal cycling mode of the Applied Biosystems 7,500 instrument was used, with an initial stage of 2 min at 50 °C followed by a 2 min period at 95 °C.

The sequences of the oligonucleotide primers for reverse transcription and amplification of representative gene transcripts in real-time quantitative PCR were designed using A plasmid Editor (ApE) software (http://biologylabs.utah.edu/jorgensen/wayned/ape/), and BLAST searches (http://blast.ncbi.nlm.nih.gov/Blast.cgi) to find unique and appropriate sequences with melting temperatures above 60 °C that had predicted low rates of homodimerization. Oligonucleotide primers were acquired from Integrated DNA Technologies, Coralville, Iowa. The sequences of oligonucleotide primers are shown in Table 1.

## Data Records

Raw FASTQ sequencing files comprised of the three biological replicates of hair cells and nsSCs, have been deposited to the NCBI Sequence Read Archive (Data Citation 2), and sample metadata can be found at the NCBI Gene Expression Omnibus (Data Citation 3) with series number of GSE101693. Individual accession numbers for each biological sample are also provided in Table 2. The most important file for readers not interested in re-analyzing the data is ‘GSE101693_Zebrafish_ Inner_Ear_Hair_Cell_Transcriptome_Table.xlsx’ (Data Citation 3).

The RPKM gene expression values of three biological replicates of hair cells and nsSCs are included in sheet 1 of ‘zFishhaircell.xlsx’ (Data Citation 4). RNA-seq raw data of zebrafish glia (Data Citation 5, Data Citation 6 and Data Citation 7) and liver cells (Data Citation 8), obtained from previously published studies^33,34^, were normalized to our datasets and reanalyzed using CLC Genomics Workbench software. The gene expression RPKM values of glia and liver cells are also included in sheet 1 of ‘zFishhaircell.xlsx’ (Data Citation 4). These data can be used for comparison with the expression pattern of hair cells and nsSCs.

## Technical Validation

### RNA quality control

**Figure 2:**
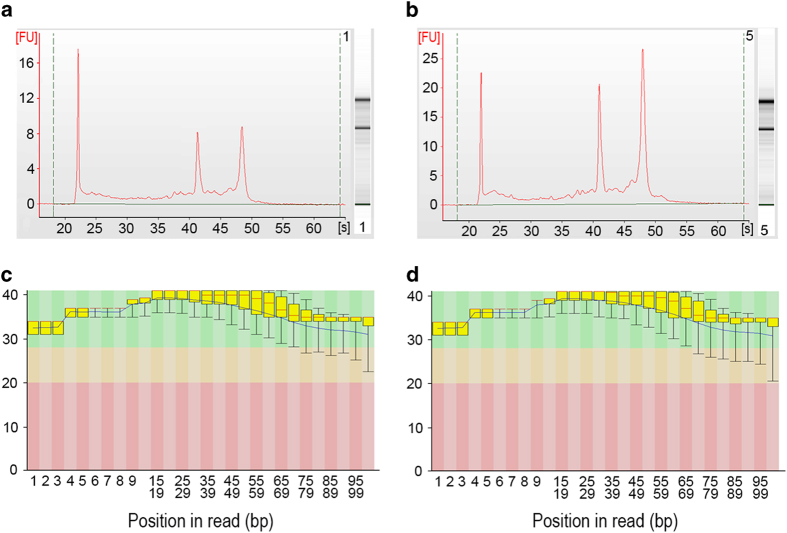
Examples of RNA-isolation quality results and Phred scores of RNA-sequencing reads. (**a**) RNA quality for hair cells. (**b**) RNA quality for nsSCs. (**c**) Phred scores for one of the sequenced hair cell samples. (**d**) Phred scores for one of the sequenced nsSC samples.

Samples of isolated RNA were analyzed for quality of intact RNA and concentration to determine their suitability for RNA-sequencing using an Agilent 2,100 BioAnalyzer. Figure 2a,b represent two examples of quality results of isolated RNA of the hair cells and nsSCs. Samples were considered to be of high quality if the ribosomal peaks had minimal (<5% of the total peak area) shoulders. All of the samples selected for RNA for sequencing met this criterion.

### Sequencing accuracy

The quality of the reads is often evaluated using the Illumina Basespace bioinformatic cloud-computing interface (https://basespace.illumina.com/ home/index). The FastQC app (version 1.0.0) on the Illumina cloud computing interface examined the quality of the reads by comparing the read signals to the probability of accurate base-reading. This number is called the Phred quality score^35^, with a score of 40 indicating a 99.99% accuracy of the base call, a score of 30 having an accuracy of 99.9%, and a score of 20 with an accuracy of 99%. The quality of the reads of our fastq.gz files generated from RNA-sequencing were analyzed for base-reading accuracy. A mean Phred score above 28, indicative of high accuracies of the correct base at a given nucleotide in the sequence, was used as the high-quality cutoff. Examples of these analyses are shown in Fig. 2c,d. All of the sequencing runs exceeded that mean, which suggests that the RNA-sequencing performed was of high quality and unambiguous.

### Reproducibility of biological samples

**Figure 3:**
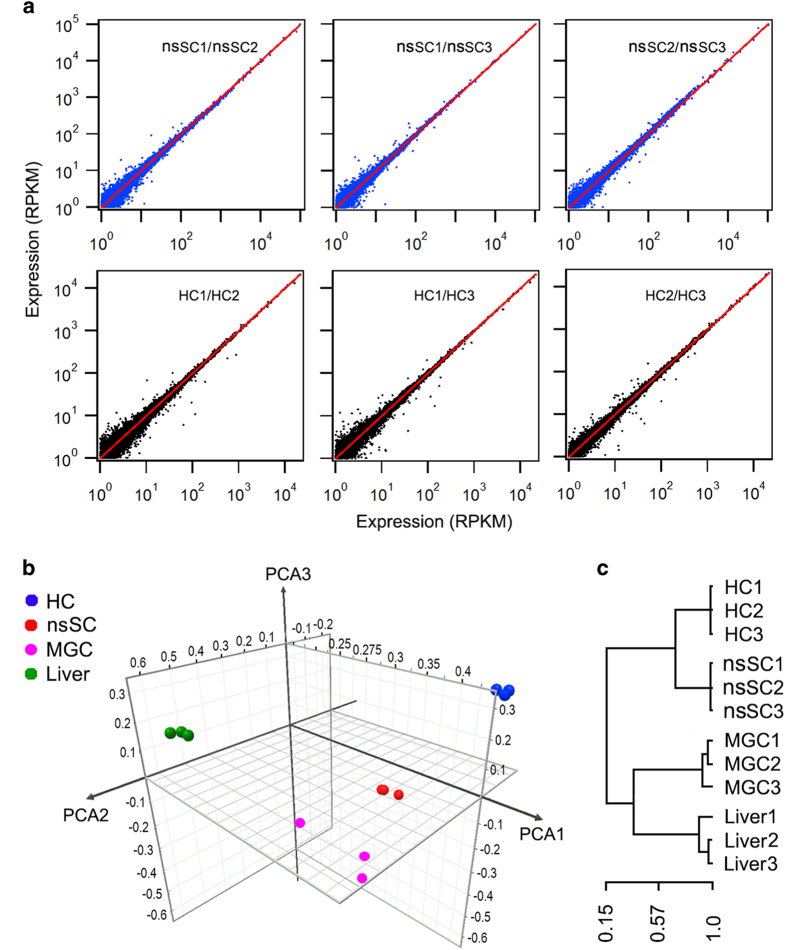
Reproducibility of biological replicates and gene expression hierarchy of different cell populations from adult zebrafish. (**a**) Correlation coefficient of biological replicates. Replicate 1 of hair cells and replicate 1 of nsSCs were separately used as reference (red lines). (**b**) PCA analysis of the gene expression profiles of hair cells and nsSCs compared with microglia (MGC)^33^ and liver cells^34^, all from adult zebrafish. The top five expressed genes in PCA analysis were: s100s, mt-co2, AC024175.9, pvalb9, mt-co3 for hair cells; mt-co2, mt-co3, AC024175.9, AC024175.4, mt-cyb for nsSCs; AC024175.4, mt-co2, mt-co1, mt-co3, tmsb4x for microglial cells; and, apoa1b, apoa2, tfa, fabp10a, and apoc1l for liver cells, respectively. (**c**) Clustering analysis of gene expression of hair cells, nsSCs, MGC, and liver cells.

We used correlation coefficient to examine reproducibility of three replicates of hair cells and nsSCs. Figure 3a show six plots of comparison between replicates from the same population of cells. As shown, the data points are all concentrated near the line (replicate 1) with small deviation. The mean correlation coefficient between hair cell replicates is 0.9984±0.0003 (mean±s.d.), while the mean correlation coefficient between nsSC replicates is 0.994±0.0045, suggesting that the results were highly reproducible.

Gene clustering analysis was also used to examine reproducibility of biological replicates and hierarchy of gene expression of different cell populations. Figure 3b shows Principal Component Analysis (PCA) of the gene expression profiles of adult zebrafish hair cells, nsSCs, microglia, and liver cells. As shown, three biological replicates of hair cells are highly reproducible as the data points are clustered all together with small variability. The three replicates of nsSCs are also highly reproducible. However, PCA analysis shows that hair cells and nsSCs are separated by a large distance, suggesting that their gene expression profiles are quite different. The gene expression profiles of microglia and liver cells are even more distinct from that of hair cells, as both of them are further away from hair cells in the graph. Gene clustering analysis displayed in Fig. 3c shows the similar results as demonstrated in PCA analysis.

### qPCR

**Figure 4:**
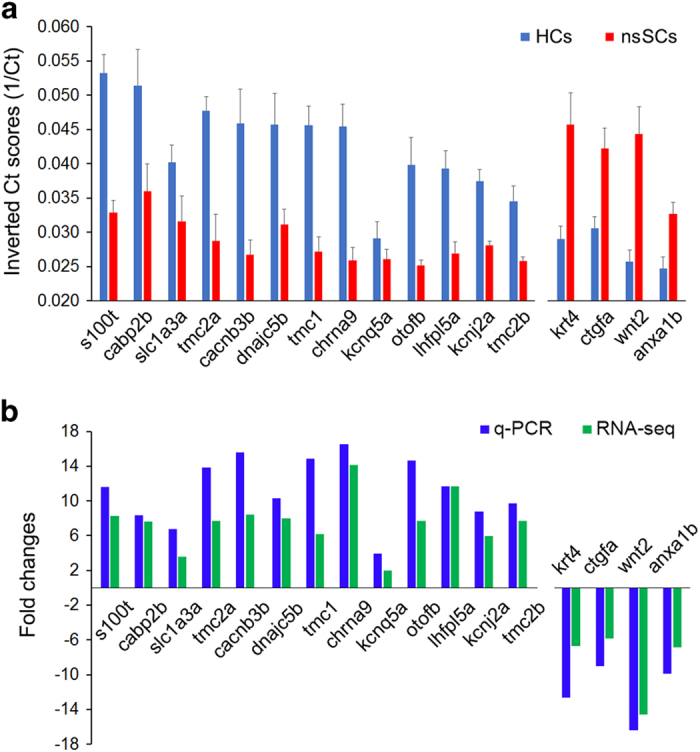
Q-PCR validation of differential expression of 17 genes. (**a**) Comparison of the expression of 18 genes in hair cells and nsSCs using q-PCR. The difference in the inverted Ct scores for each gene between hair cells and nsSCs was statistically significant (*P*≤0.01, *n*=3). Note that the ordinate is the inverse of the number of PCR cycles necessary to reach fluorescence threshold. So even a small difference (such as 0.01) reflects a large difference between the two cell populations^36^. (**b**) Fold differences in expression of these 17 genes between hair cells and nsSCs using q-PCR and RNA-seq. Positive values indicate higher gene expression in hair cells while negative values indicate higher expression values in nsSCs. Fold differences are all calculated in log2 base.

We separately prepared three biological replicates of hair cells and nsSCs from 15 additional zebrafish for qPCR to validate the expression of 17 genes, with 13 being highly expressed in hair cells and 4 highly expressed in nsSCs. The expression values were all normalized to the cycle threshold (Ct) value of *actb2*, which was used as the reference gene for hair cells and nsSCs. The normalized Ct numbers were then inverted (1/Ct)^36^ and graphed in Fig. 4a. We compared the patterns of differential expression of these genes between hair cells and nsSCs using expression values from qPCR and RNA-seq. As shown, 13 genes that were differentially expressed in hair cells based on RNA-seq analysis show larger inverted Ct scores in hair cells than in nsSCs, suggesting that their expression in hair cells is greater than in nsSCs. In contrast, four genes that had higher expression in nsSCs than in hair cells in RNA-seq analysis also show larger inverted Ct scores in nsSCs than in hair cells (Fig. 4a). Figure 4b shows such a comparison after the expression values were normalized to fold changes. Although the level of expression is normally not comparable between the two techniques (due to different amplification and quantification procedures), the trend of differential expression of these genes is consistent with our RNA-seq data.

### Validation by comparison with published studies

Several studies have examined the gene expression profiles of hair cells from inner ear and lateral line from larval or adult zebrafish^16,18,20,21^. Enriched genes in hair cells and nsSCs identified in these studies can be used for comparison with enriched genes identified in current study. Table 3 shows the RPKM values of enriched transcripts of hair cells and nsSCs from current study. A list of enriched transcripts in hair cells (top 21 transcripts in Table 3) and nsSCs (bottom 5 transcripts in the table) was compiled from previous studies^16,18,21^. The differential expression of these transcripts in hair cells or nsSCs was validated by *in situ* hybridization in those studies. The differential expression pattern shown in our dataset is highly consistent with the pattern demonstrated by *in situ* hybridization in previous studies. Comparison with published data offers another way for validation of our dataset. In Table 4, the top 30 enriched zebrafish hair cells transcripts and the expression value of their mammalian orthologs in the adult mouse inner ear are presented. The expression values of mouse inner hair cell and outer hair cell were from our previous study^24^. Since the expression values from zebrafish and mouse hair cells cannot be normalized for direct comparison, the abundance ranking of the mammalian orthologs detected in adult inner hair cells and outer hair cells is also presented in Table 4 for comparison. Note that except for three genes (*Ocm*, *Atp1b2* and *Hspa8*), most genes or transcripts that are highly expressed in zebrafish hair cells are only expressed at modest to low levels in adult mouse hair cells. Six of the zebrafish genes have no known mouse ortholog, and two of the genes (*Ocm* and *Gm42674*) have at least two zebrafish orthologs for a single mouse gene.

## Usage Notes

Transcriptome analysis is often used to identify differentially expressed genes that may underpin unique biological properties of cells. The transcriptomes of hair cells and nsSCs presented in this study can be used to identify enriched genes or transcripts of hair cells and nsSCs in adult zebrafish inner ear. Sheet 2 of ‘zFishhaircell.xlsx’ (Data Citation 4) presents the uniquely (in red fonts) and differentially (in blue fonts) expressed genes in hair cells and nsSCs. Uniquely expressed genes were those whose expression levels were below background in only one cell type, whereas differentially expressed genes were categorized as those whose RPKM values were above 0.1 and at least 2-fold different between the two cell types with statistical significance (Student’s *t*-test with *P*≤0.05). These uniquely and differentially expressed genes may provide valuable information to understand unique structures and function of hair cells and nsSCs in the adult zebrafish inner ear.

## Additional information

**How to cite this article:** Barta, C. L. *et al.* RNA-seq transcriptomic analysis of adult zebrafish inner ear hair cells. *Sci. Data* 5:180005 doi: 10.1038/sdata.2018.5 (2018).

**Publisher’s note:** Springer Nature remains neutral with regard to jurisdictional claims in published maps and institutional affiliations.

## Supplementary Material



## Figures and Tables

**Table 1:** qPCR Primer sequences.

**Gene name**	**Forward Primer Sequence 5-3**	**Reverse Primer Sequence 5-3**
s100t	TGGGAATGAGGGTGACAAAT	TCAITCGCTGGTCATGTGTT
cabp2b	AGCTTCTCTCAGCTCITCAATCTC	GGCAAAGTGAAACGGGCCTGTC
otofb	GGCGCTTCATITATCCTTTCGAC	GACGAGGTGCCGGATTGCCTTTAGC
tmc2a	AGCCAATCCTAATGCACGGG	ACACATCACTGCCGTCTAGG
dnajc5b	AGCAACCCGACTCCATTAGC	GTCAAGGCCCAGAACTTGGTA
cacnb3b	CGGAAGAAAAGCAGAAGCGACG	CAGAGGCAATGGCACAGACAG
chrna9	CATGGAGAGTGGTGATCTTTC	GCAGGACTGTGTACGTTATG
lhfpl5a	AGGACAAACTGCTGCCAGAGGAC	CITCAGAAAGTGAATGCGTTCG
slc1a3a	TGTCGTATCGGGAGGTGAAG	ACTGTCCAGAGCTGCCATTC
tmc2b	TCACGGAGACAATAGACAAGGA	AATAGCCAACCACACCATGAG
kcnj2a	ATCAGAGGAGGACGGTATGAAGTTG	CGCATGTGGTGAAGATGTCAGC
tmc1	CACGCTACTCATCACCGGCA	GCCTTGCATGTGGTTGACCG
kcnq5a	AAACTCCAATCTCACGGCGG	TCACAGTTCCTGACAAGTGC
ctgfa	GCACAGATGGTCGCTGTTGCACTC	CCAAGAATAGTCAAGTCAGCAC
krt4	TACCATTCATGTTCAGCAGACCTC	CCTGATCCAGTGAAACCATAGTAC
wnt2	ACACATCAAGAGTGAGTCGCACAAC	GAAGITGCTGGTCATGTTGTGTACG
anxa1b	TCAGGCGGCCATTCAGAAAGAAAC	CACAGCCAGCAGTTTCAATACAAG
Oligonucleotide primers were used to examine the expression of HC and nsSC transcripts using qPCR.		

**Table 2:** Description of the data generated from RNA-sequencing of individually collected zebrafish hair cells and surrounding cells deposited in the Gene Expression Omnibus (GSE101693).

**Organism**	**Sample**	**Replicate**	**Analysis type**	**GEO number**
*Danio rerio*	1,000GFP^+^ inner ear HCs	Biological Replicate 1	RNA-Sequencing (paired-end)	GSM2712279
*Danio rerio*	1,000GFP^+^ inner ear HCs	Biological Replicate 2	RNA-Sequencing (paired-end)	GSM2712280
*Danio rerio*	1,000GFP^+^ inner ear HCs	Biological Replicate 3	RNA-Sequencing (paired-end)	GSM2712281
*Danio rerio*	1,000GFP^−^ inner ear nsSCs	Biological Replicate 1	RNA-Sequencing (paired-end)	GSM2712282
*Danio rerio*	1,000GFP^−^ inner ear nsSCs	Biological Replicate 2	RNA-Sequencing (paired-end)	GSM2712283
*Danio rerio*	1,000GFP^−^ inner ear nsSCs	Biological Replicate 3	RNA-Sequencing (paired-end)	GSM2712284

**Table 3:** Enriched transcripts detected in RNA-seq and validated by *in situ* hybridization in previous studies^16,21^.

**Gene Name**	**HC RPKM**	**Std. Dev.**	**nsSC RPKM**	**Std. Dev.**
**anxa5a**	2053.1	43.1	2.5	0.3
**baiap2l2b**	202.7	13.6	0.7	0.1
**cabp2b**	5795.6	98.5	29.0	1.0
**cd164l2**	3945.1	8.3	8.4	1.1
**CD37**	265.1	10.7	2.6	0.1
**chrna9**	182.0	8.1	0.0	0.0
**dnajc5b**	490.0	12.4	1.1	0.3
**fam188b2**	116.4	2.5	0.0	0.0
**myo15ab**	19.4	0.6	0.0	0.0
**myo1ha**	23.0	0.7	0.0	0.0
**myo6b**	71.1	2.3	0.4	0.1
**pcsk5a**	79.2	2.7	0.1	0.0
**s100s**	21338.4	182.4	87.4	2.5
**s100t**	10340.0	239.6	33.5	4.3
**si:dkey-229d2.6**	5.7	2.2	0.3	0.1
**si:dkey-229d2.7**	502.4	8.7	3.2	1.4
**si:dkeyp-110e4.11**	1138.4	74.4	1.6	0.2
**si:rp71-68n21.12**	1176.2	37.0	5.5	0.3
**slc17a8**	827.0	21.5	0.5	0.1
**tekt3**	212.0	5.0	1.9	0.2
**tmc2a**	578.9	3.7	2.8	0.0
**krt4**	3.7	0.7	379.0	9.5
**nfe2l2a**	10.1	0.3	49.9	1.9
**si:dkey-247k7.2**	0.0	0.0	68.2	14.2
**tnks1bp1**	1.3	0.2	10.5	0.9
**zgc:198419**	498.1	21.7	123.8	8.5

**Table 4:** Expression values of top 30 enriched zebrafish hair cell genes and the corresponding mouse orthologs in adult mouse hair cells.

**zebrafish**				**Mice (from Liu** ***et al***.)^24^			
**Gene Name**	**HC average**	**Ensembl Gene ID**	**Gene Name**	**OHC average**	**Ranking in OHCs**	**IHC average**	**Ranking in IHCs**
s100s	21338.4	ENSMUSG00000001023	s100a5	4.6	23598	3.7	24612
pvalb9	15432.5	ENSMUSG00000029618	Ocm	4179.6	6	865.6	236
s100t	10340.0	ENSMUSG00000001023	s100a5	4.6	23598	3.7	24612
mb	9989.2	ENSMUSG00000018893	Mb	15.9	14210	15.5	13211
pvalb8	7101.5	ENSMUSG00000029618	Ocm	4179.6	6	865.6	236
ckbb	6838.0	ENSMUSG00000001270	Ckb	364.0	575	573.7	407
cabp2b	5795.6	ENSMUSG00000024857	Cabp2	308.2	771	351.6	717
fosab	5458.7	ENSMUSG00000021250	Fos	23.4	8408	31.5	7847
gapdhs	4839.3	ENSMUSG00000061099	Gapdhs	16.3	13246	20.5	10908
cd164l2	3945.1	ENSMUSG00000028865	Cd164l2	522.7	386	642.7	347
si:ch211-243g18.2	3478.3	ENSMUSG00000059169	Krt40	15.0	14364	13.8	14338
CU855484.1	3042.0	ENSMUSG00000042808	Gpx2	128.6	1932	110.6	2531
calm3a	3021.3	no ortho					
atp1b2b	2729.9	ENSMUSG00000041329	Atp1b2	273.2	836	121.5	2302
zgc:92066	2400.3	no ortho					
calm2b	2312.3	ENSMUSG00000001175	Calm1	2625.2	25	2912.5	16
rtn4rl2b	2253.3	ENSMUSG00000050896	Rtn4rl2	35.0	6733	37.8	6745
si:dkey-11c5.11	2239.3	no ortho					
calm1b	2099.8	ENSMUSG00000099269	Calm5	17.0	12836	13.4	14575
anxa5a	2053.1	ENSMUSG00000027712	Anxa5	606.3	317	1040.9	196
calm1a	2040.7	no ortho					
clta	2040.6	ENSMUSG00000028478	Clta	256.2	900	391.0	637
jun	1996.3	ENSMUSG00000052684	Jun	16.2	13311	72.2	3840
atp5o	1699.4	ENSMUSG00000022956	Atp5o	19.5	11522	16.4	12672
aldocb	1668.2	no ortho					
COX5B(1of many)	1505.9	ENSMUSG00000061518	Cox5b	45.8	5233	36.9	6875
arg2	1402.6	ENSMUSG00000021125	Arg2	50.8	4863	29.0	8399
ddx5	1391.3	ENSMUSG00000020719	Ddx5	607.6	314	645.5	344
hspa8	1369.2	ENSMUSG00000015656	Hspa8	3730.8	8	3825.9	7
cox6a1	1312.3	no ortho					
tspan13b	1271.2	ENSMUSG00000020577	Tspan13	423.4	502	855.7	238.0
